# 2-[3,5-Bis(4-meth­oxy­phen­yl)-4,5-di­hydro-1*H*-pyrazol-1-yl]-4,6-bis­(4-meth­oxy­phen­yl)pyrimidine

**DOI:** 10.1107/S1600536812030516

**Published:** 2012-07-10

**Authors:** Rajni Kant, Vivek K. Gupta, Kamini Kapoor, S. Samshuddin, B. Narayana

**Affiliations:** a X-ray Crystallography Laboratory, Post-Graduate Department of Physics and Electronics, University of Jammu, Jammu Tawi 180 006, India; b Department of Studies in Chemistry, Mangalore University, Mangalagangotri 574 199, India

## Abstract

In the title compound, C_35_H_32_N_4_O_4_, the pyrazole ring forms a dihedral angle of 15.04 (8)° with the adjacent pyrimidine ring. The pyrimidine ring forms dihedral angles of 9.95 (8) and 1.86 (7)° with its adjacent meth­oxy-substituted benzene rings, whereas the equivalent angles are 80.24 (9) and 11.55 (9)° for the pyrazole ring and its adjacent benzene rings. The crystal packing features π–π inter­actions, the centroid–centroid distance between the pyrimidine and methoxyphenyl rings being 3.604 (1) Å. The pyrazole ring is nearly planar, with a maximum deviation of 0.020 (3) Å for the –CH_2_– carbon.

## Related literature

For biological importance of substituted pyrimidines, see: Fun *et al.* (2010 ▶); Jasinski *et al.* (2010 ▶); Baktır *et al.* (2011 ▶); Samshuddin *et al.* (2011 ▶); Betz *et al.* (2012 ▶). For related literature on substituted pyrimidines and their derivatives, see: Calabresi *et al.* (1975 ▶); El-Hashash *et al.* (1993 ▶); Fun *et al.* (2012 ▶).
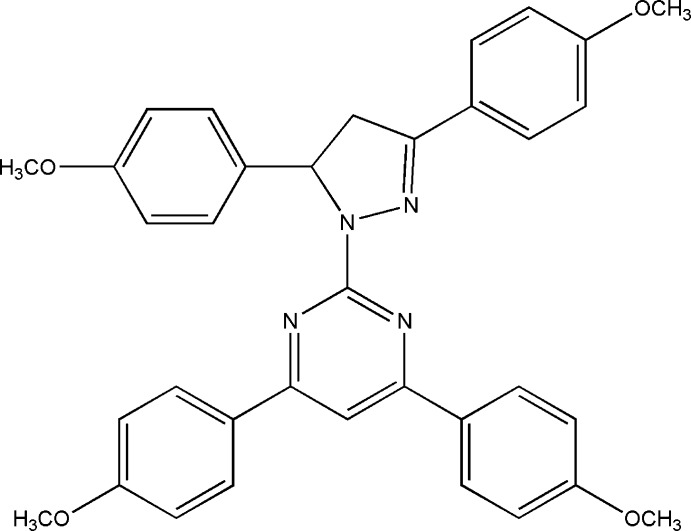


## Experimental

### 

#### Crystal data

C_35_H_32_N_4_O_4_
*M* *_r_* = 572.65
Monoclinic, 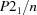

*a* = 21.637 (2) Å
*b* = 5.9532 (4) Å
*c* = 24.749 (2) Å
β = 109.519 (10)°
*V* = 3004.7 (5) Å^3^
*Z* = 4
Mo *K*α radiation
μ = 0.08 mm^−1^
*T* = 293 K
0.3 × 0.2 × 0.2 mm

#### Data collection

Agilent Xcalibur Sapphire3 diffractometer
Absorption correction: multi-scan (*CrysAlis PRO*; Oxford Diffraction, 2010 ▶) *T*_min_ = 0.887, *T*_max_ = 1.000
12486 measured reflections
5824 independent reflections
3423 reflections with *I* > 2σ(*I*)
*R*_int_ = 0.028

#### Refinement

*R*[*F* ^2^ > 2σ(*F* ^2^)] = 0.052
*wR*(*F* ^2^) = 0.145
*S* = 1.05
5824 reflections
393 parameters
H-atom parameters constrained
Δρ_max_ = 0.16 e Å^−3^
Δρ_min_ = −0.15 e Å^−3^

### 

Data collection: *CrysAlis PRO* (Oxford Diffraction, 2010 ▶); cell refinement: *CrysAlis PRO*; data reduction: *CrysAlis PRO*; program(s) used to solve structure: *SHELXS97* (Sheldrick, 2008 ▶); program(s) used to refine structure: *SHELXL97* (Sheldrick, 2008 ▶); molecular graphics: *ORTEP-3* (Farrugia, 1997 ▶); software used to prepare material for publication: *PLATON* (Spek, 2009 ▶).

## Supplementary Material

Crystal structure: contains datablock(s) I, global. DOI: 10.1107/S1600536812030516/bh2441sup1.cif

Structure factors: contains datablock(s) I. DOI: 10.1107/S1600536812030516/bh2441Isup2.hkl

Supplementary material file. DOI: 10.1107/S1600536812030516/bh2441Isup3.cml

Additional supplementary materials:  crystallographic information; 3D view; checkCIF report

## Figures and Tables

**Fig. 1.**
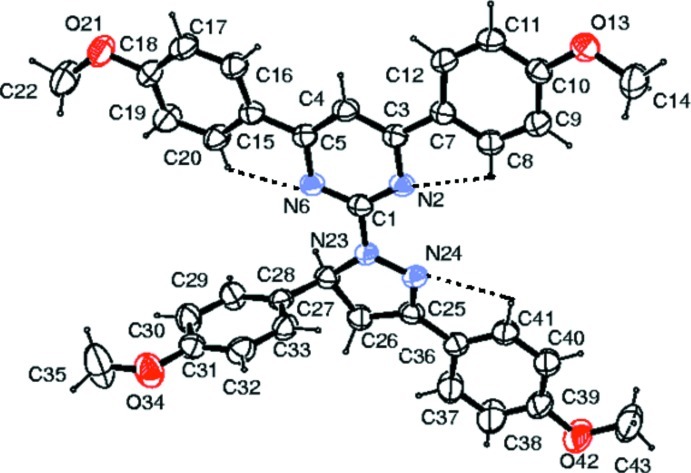
ORTEP view of the molecule with thermal ellipsoids drawn at the 40% probability level. H atoms are shown as small spheres of arbitrary radii.

**Fig. 2.**
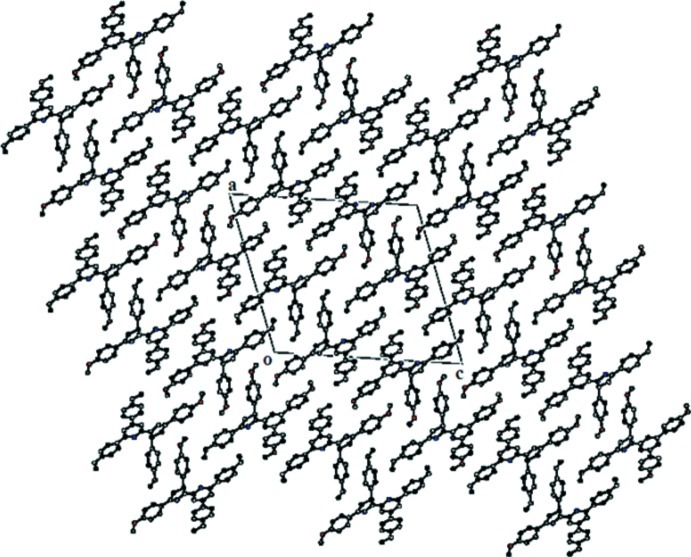
The packing arrangement of molecules viewed down the b-axis.

## supplementary crystallographic information

### Comment

The importance of pyrimidines and analogous compounds in pharmaceutical and biological fields is well known. Some substituted pyrimidines and their derivatives have been reported to possess antimicrobial and antifungal activities (El-Hashash *et al.*, 1993). It has incidental antiviral activity against herpes and vaccinia infections (Calabresi *et al.*, 1975). With the development of clinically useful pyrimidine based antitumor and antiviral drugs there has been noticeable interest in synthetic manipulations of pyrimidines. In view of the biological importance of pyrimidines and in continuation of work on synthesis of various derivatives of chalcone (Samshuddin *et al.*, 2011; Fun *et al.*, 2010; Jasinski *et al.*, 2010; Baktır *et al.*, 2011; Betz *et al.*, 2012), the title compound is prepared and its crystal structure is reported.

The molecule comprises of the pyrimidine ring, pyrazole ring, and four methoxy substituted benzene rings. All bond lengths and angles are normal and correspond to those observed in related structure (Fun *et al.*, 2012). The six bond lengths in the pyrimidine ring lie in the range 1.337 (2)–1.396 (3) Å. The pyrimidine ring and pyrazole ring are individually planar with maximum deviations from the respective least-squares planes of: 0.010 (2) Å for C1 and 0.021 (3) Å for C26. Three intramolecular interactions C8—H8···N2, C20—H20···N6 and C41—H41···N24 are observed which lock the molecular conformation and thus eliminating conformational flexibility (Fig. 1). The pyrazole ring forms a dihedral angle of 15.04 (8)° with the adjacent pyrimidine ring (maximum deviation = -0.0176 (2) Å at atom C27). The pyrimidine ring forms dihedral angles of 9.95 (8) and 1.86 (7)° with its adjacent methoxy-substituted benzene rings (C15···C20 & C7···C12, respectively), whereas for pyrazole ring these angles are 80.24 (9) and 11.55 (9)° (C28···C33 & C36···C41, respectively). Molecules in the crystal are packed together to form layers, which appear to be extending diagonally along the *ac* plane (Fig. 2). Examination of non-bonded contacts reveals no classical intermolecular hydrogen bonds. The crystal structure is stabilized by π–π interaction between the pyrimidine ring of the molecule at (*x*, *y*, *z*) and benzene ring (C7···C12) at (1 - *x*, -*y*, 1 - *z*) [centroid separation = 3.604 (1) Å, interplanar spacing = 3.45 Å and centroid shift = 1.06 Å].

### Experimental

A mixture of 4,4'-dimethoxy chalcone (2.68 g, 0.01 mol) and amino guanidine hydrochloride (0.065 g, 0.005 mol) in 25 ml e thanol was refluxed for 24 hrs in the presence of sodium ethoxide (2 ml). The reaction mixture was cooled to room temperature and refrigerated overnight. The solid product obtained was filtered and recrystallized from ethanol, affording a yellow powder. Single crystals were grown from DMF by slow evaporation method and the yield of the compound was 64% (m.p. 502 K).

### Refinement

All H atoms were positioned geometrically and were treated as riding on their parent C atoms, with C—H distances of 0.93–0.98 Å and with *U*_iso_(H) = 1.2*U*_eq_(C) or 1.5*U*_eq_(methyl C).

### Crystal data

**Table:**

C_35_H_32_N_4_O_4_	*F*(000) = 1208
*M**_r_* = 572.65	*D*_x_ = 1.266 Mg m^−^^3^
Monoclinic, *P*2_1_/*n*	Melting point: 502 K
Hall symbol: -P 2yn	Mo *K*α radiation, λ = 0.71073 Å
*a* = 21.637 (2) Å	Cell parameters from 4664 reflections
*b* = 5.9532 (4) Å	θ = 3.1–32.2°
*c* = 24.749 (2) Å	µ = 0.08 mm^−^^1^
β = 109.519 (10)°	*T* = 293 K
*V* = 3004.7 (5) Å^3^	Needle, white
*Z* = 4	0.3 × 0.2 × 0.2 mm

### Data collection

**Table:**

Agilent Xcalibur Sapphire3 diffractometer	5824 independent reflections
Radiation source: fine-focus sealed tube	3423 reflections with *I* > 2σ(*I*)
Graphite monochromator	*R*_int_ = 0.028
Detector resolution: 16.1049 pixels mm^-1^	θ_max_ = 26.0°, θ_min_ = 3.1°
ω scan	*h* = −26→22
Absorption correction: multi-scan (*CrysAlis PRO*; Oxford Diffraction, 2010)	*k* = −7→7
*T*_min_ = 0.887, *T*_max_ = 1.000	*l* = −30→29
12486 measured reflections	

### Refinement

**Table:**

Refinement on *F*^2^	Secondary atom site location: difference Fourier map
Least-squares matrix: full	Hydrogen site location: inferred from neighbouring sites
*R*[*F*^2^ > 2σ(*F*^2^)] = 0.052	H-atom parameters constrained
*wR*(*F*^2^) = 0.145	*w* = 1/[σ^2^(*F*_o_^2^) + (0.0751*P*)^2^] where *P* = (*F*_o_^2^ + 2*F*_c_^2^)/3
*S* = 1.05	(Δ/σ)_max_ = 0.001
5824 reflections	Δρ_max_ = 0.16 e Å^−^^3^
393 parameters	Δρ_min_ = −0.15 e Å^−^^3^
0 restraints	Extinction correction: *SHELXL97* (Sheldrick, 2008), Fc^*^=kFc[1+0.001xFc^2^λ^3^/sin(2θ)]^-1/4^
0 constraints	Extinction coefficient: 0.0036 (7)
Primary atom site location: structure-invariant direct methods	

### Fractional atomic coordinates and isotropic or equivalent isotropic displacement parameters (Å^2^)

**Table:**

	*x*	*y*	*z*	*U*_iso_*/*U*_eq_	
C1	0.94094 (10)	0.2692 (3)	0.65771 (9)	0.0460 (5)	
N2	0.97976 (8)	0.4493 (3)	0.66528 (7)	0.0468 (4)	
C3	0.97863 (9)	0.5581 (3)	0.61747 (8)	0.0435 (5)	
C4	0.93798 (10)	0.4855 (3)	0.56368 (9)	0.0501 (5)	
H4	0.9372	0.5613	0.5306	0.060*	
C5	0.89887 (9)	0.2986 (3)	0.56049 (8)	0.0443 (5)	
N6	0.89964 (8)	0.1872 (3)	0.60806 (7)	0.0475 (4)	
C7	1.02191 (9)	0.7565 (3)	0.62675 (8)	0.0440 (5)	
C8	1.05920 (11)	0.8194 (4)	0.68218 (9)	0.0607 (6)	
H8	1.0567	0.7329	0.7127	0.073*	
C9	1.09970 (12)	1.0049 (4)	0.69371 (9)	0.0619 (6)	
H9	1.1234	1.0428	0.7314	0.074*	
C10	1.10489 (10)	1.1338 (3)	0.64922 (9)	0.0486 (5)	
C11	1.06833 (11)	1.0755 (4)	0.59376 (9)	0.0590 (6)	
H11	1.0710	1.1626	0.5634	0.071*	
C12	1.02815 (10)	0.8910 (3)	0.58281 (9)	0.0547 (6)	
H12	1.0044	0.8546	0.5450	0.066*	
O13	1.14306 (8)	1.3213 (3)	0.65618 (6)	0.0673 (5)	
C14	1.17755 (15)	1.3895 (5)	0.71345 (11)	0.0891 (9)	
H14A	1.1475	1.4002	0.7344	0.134*	
H14B	1.1975	1.5333	0.7131	0.134*	
H14C	1.2109	1.2810	0.7315	0.134*	
C15	0.85339 (10)	0.2065 (3)	0.50591 (8)	0.0467 (5)	
C16	0.83848 (11)	0.3217 (4)	0.45394 (9)	0.0578 (6)	
H16	0.8589	0.4583	0.4527	0.069*	
C17	0.79427 (12)	0.2366 (4)	0.40474 (10)	0.0656 (7)	
H17	0.7852	0.3163	0.3707	0.079*	
C18	0.76293 (11)	0.0351 (4)	0.40476 (9)	0.0565 (6)	
C19	0.77751 (12)	−0.0839 (4)	0.45515 (10)	0.0634 (6)	
H19	0.7574	−0.2215	0.4558	0.076*	
C20	0.82231 (11)	0.0022 (3)	0.50497 (9)	0.0589 (6)	
H20	0.8318	−0.0796	0.5388	0.071*	
O21	0.71861 (9)	−0.0292 (3)	0.35312 (7)	0.0772 (5)	
C22	0.68206 (15)	−0.2275 (5)	0.35211 (12)	0.0921 (9)	
H22A	0.7113	−0.3538	0.3622	0.138*	
H22B	0.6511	−0.2491	0.3143	0.138*	
H22C	0.6591	−0.2140	0.3791	0.138*	
N23	0.94146 (8)	0.1510 (3)	0.70559 (7)	0.0532 (5)	
C27	0.90703 (10)	−0.0654 (3)	0.70347 (8)	0.0497 (5)	
H27	0.9161	−0.1646	0.6754	0.060*	
C26	0.94213 (11)	−0.1561 (4)	0.76440 (9)	0.0589 (6)	
H26A	0.9112	−0.1843	0.7844	0.071*	
H26B	0.9658	−0.2936	0.7633	0.071*	
C25	0.98826 (10)	0.0304 (3)	0.79219 (9)	0.0512 (5)	
N24	0.98766 (9)	0.1968 (3)	0.75857 (7)	0.0516 (4)	
C28	0.83408 (10)	−0.0386 (3)	0.68865 (8)	0.0466 (5)	
C29	0.79248 (11)	−0.1969 (4)	0.65509 (10)	0.0594 (6)	
H29	0.8099	−0.3149	0.6402	0.071*	
C30	0.72521 (12)	−0.1855 (4)	0.64285 (10)	0.0668 (7)	
H30	0.6980	−0.2946	0.6200	0.080*	
C31	0.69907 (11)	−0.0116 (4)	0.66471 (9)	0.0562 (6)	
C32	0.73989 (12)	0.1499 (4)	0.69814 (9)	0.0603 (6)	
H32	0.7223	0.2685	0.7127	0.072*	
C33	0.80649 (11)	0.1356 (3)	0.70990 (9)	0.0550 (6)	
H33	0.8336	0.2452	0.7326	0.066*	
O34	0.63314 (8)	0.0149 (3)	0.65443 (8)	0.0832 (6)	
C35	0.59055 (14)	−0.1475 (6)	0.61983 (16)	0.1181 (12)	
H35A	0.5895	−0.1317	0.5809	0.177*	
H35B	0.5472	−0.1264	0.6215	0.177*	
H35C	0.6059	−0.2950	0.6335	0.177*	
C36	1.03203 (11)	0.0316 (3)	0.85193 (9)	0.0538 (6)	
C37	1.02482 (14)	−0.1251 (5)	0.89076 (11)	0.0897 (9)	
H37	0.9925	−0.2346	0.8786	0.108*	
C38	1.06520 (16)	−0.1204 (5)	0.94740 (11)	0.1114 (13)	
H38	1.0594	−0.2262	0.9729	0.134*	
C39	1.11359 (13)	0.0376 (4)	0.96645 (10)	0.0758 (7)	
C40	1.12168 (12)	0.1934 (4)	0.92856 (10)	0.0691 (7)	
H40	1.1545	0.3014	0.9409	0.083*	
C41	1.08110 (12)	0.1895 (4)	0.87217 (10)	0.0639 (6)	
H41	1.0870	0.2966	0.8470	0.077*	
O42	1.15190 (11)	0.0266 (4)	1.02335 (8)	0.1112 (8)	
C43	1.20421 (18)	0.1810 (5)	1.04362 (12)	0.1177 (13)	
H43A	1.2342	0.1608	1.0229	0.177*	
H43B	1.2267	0.1557	1.0837	0.177*	
H43C	1.1873	0.3314	1.0380	0.177*	

### Atomic displacement parameters (Å^2^)

**Table:**

	*U*^11^	*U*^22^	*U*^33^	*U*^12^	*U*^13^	*U*^23^
C1	0.0467 (12)	0.0489 (11)	0.0462 (12)	0.0054 (10)	0.0206 (10)	0.0098 (9)
N2	0.0513 (10)	0.0458 (9)	0.0446 (10)	−0.0002 (8)	0.0177 (8)	0.0078 (8)
C3	0.0433 (11)	0.0430 (11)	0.0447 (12)	0.0081 (9)	0.0155 (9)	0.0088 (9)
C4	0.0552 (13)	0.0519 (12)	0.0435 (12)	0.0034 (10)	0.0169 (10)	0.0124 (9)
C5	0.0438 (12)	0.0453 (11)	0.0441 (12)	0.0104 (9)	0.0149 (9)	0.0071 (9)
N6	0.0482 (10)	0.0526 (9)	0.0419 (10)	0.0018 (8)	0.0155 (8)	0.0061 (8)
C7	0.0402 (11)	0.0468 (11)	0.0455 (12)	0.0065 (9)	0.0150 (9)	0.0073 (9)
C8	0.0665 (15)	0.0661 (14)	0.0468 (13)	−0.0097 (12)	0.0153 (11)	0.0152 (11)
C9	0.0662 (15)	0.0674 (14)	0.0475 (13)	−0.0134 (12)	0.0127 (11)	0.0052 (11)
C10	0.0464 (12)	0.0481 (12)	0.0526 (13)	0.0012 (9)	0.0182 (10)	0.0058 (10)
C11	0.0711 (15)	0.0593 (13)	0.0480 (13)	−0.0093 (12)	0.0218 (11)	0.0112 (10)
C12	0.0629 (14)	0.0584 (13)	0.0411 (12)	−0.0061 (11)	0.0152 (10)	0.0063 (10)
O13	0.0763 (11)	0.0639 (10)	0.0612 (10)	−0.0188 (8)	0.0222 (9)	0.0029 (7)
C14	0.104 (2)	0.0834 (17)	0.0679 (18)	−0.0364 (16)	0.0122 (16)	−0.0077 (14)
C15	0.0478 (12)	0.0482 (11)	0.0442 (12)	0.0069 (10)	0.0155 (10)	0.0061 (9)
C16	0.0634 (14)	0.0560 (12)	0.0500 (13)	−0.0029 (11)	0.0134 (11)	0.0090 (11)
C17	0.0752 (16)	0.0707 (15)	0.0461 (14)	0.0014 (13)	0.0137 (12)	0.0164 (11)
C18	0.0568 (14)	0.0633 (14)	0.0434 (13)	0.0049 (11)	0.0089 (11)	0.0014 (10)
C19	0.0789 (17)	0.0503 (12)	0.0550 (14)	−0.0058 (12)	0.0145 (12)	0.0029 (11)
C20	0.0714 (16)	0.0534 (12)	0.0470 (13)	0.0014 (11)	0.0134 (11)	0.0087 (10)
O21	0.0823 (12)	0.0822 (11)	0.0512 (10)	−0.0099 (10)	0.0010 (9)	0.0016 (8)
C22	0.105 (2)	0.0825 (19)	0.0681 (18)	−0.0162 (17)	0.0016 (16)	−0.0098 (14)
N23	0.0575 (11)	0.0594 (10)	0.0409 (10)	−0.0143 (9)	0.0140 (9)	0.0064 (8)
C27	0.0565 (13)	0.0490 (12)	0.0466 (12)	−0.0042 (10)	0.0212 (10)	0.0023 (9)
C26	0.0574 (14)	0.0606 (13)	0.0562 (14)	−0.0063 (11)	0.0155 (11)	0.0113 (11)
C25	0.0530 (13)	0.0541 (12)	0.0490 (13)	−0.0014 (10)	0.0204 (10)	0.0089 (10)
N24	0.0581 (11)	0.0560 (10)	0.0401 (10)	−0.0057 (9)	0.0158 (8)	0.0038 (8)
C28	0.0556 (13)	0.0496 (11)	0.0373 (11)	−0.0038 (10)	0.0191 (10)	0.0021 (9)
C29	0.0582 (15)	0.0548 (12)	0.0684 (15)	−0.0033 (11)	0.0252 (12)	−0.0180 (11)
C30	0.0598 (15)	0.0649 (14)	0.0764 (17)	−0.0112 (12)	0.0236 (13)	−0.0224 (12)
C31	0.0538 (14)	0.0636 (13)	0.0544 (13)	0.0031 (11)	0.0223 (11)	−0.0043 (11)
C32	0.0676 (16)	0.0597 (13)	0.0557 (14)	0.0069 (12)	0.0234 (12)	−0.0110 (11)
C33	0.0622 (15)	0.0533 (12)	0.0472 (13)	−0.0055 (11)	0.0153 (11)	−0.0088 (10)
O34	0.0555 (11)	0.0960 (13)	0.0997 (14)	0.0022 (9)	0.0278 (10)	−0.0225 (10)
C35	0.0585 (18)	0.125 (3)	0.166 (3)	−0.0159 (18)	0.032 (2)	−0.044 (2)
C36	0.0547 (13)	0.0593 (13)	0.0459 (13)	−0.0015 (11)	0.0149 (10)	0.0080 (10)
C37	0.092 (2)	0.0934 (19)	0.0640 (17)	−0.0369 (16)	0.0006 (15)	0.0306 (14)
C38	0.122 (3)	0.125 (2)	0.0598 (18)	−0.052 (2)	−0.0063 (18)	0.0448 (17)
C39	0.0825 (19)	0.0872 (17)	0.0469 (14)	−0.0161 (15)	0.0075 (13)	0.0133 (13)
C40	0.0721 (16)	0.0742 (15)	0.0548 (15)	−0.0168 (13)	0.0128 (12)	0.0081 (12)
C41	0.0732 (16)	0.0665 (14)	0.0499 (14)	−0.0136 (13)	0.0177 (12)	0.0128 (11)
O42	0.1200 (17)	0.1355 (17)	0.0529 (11)	−0.0441 (14)	−0.0047 (11)	0.0269 (11)
C43	0.137 (3)	0.123 (2)	0.0618 (19)	−0.050 (2)	−0.0081 (19)	0.0032 (17)

### Geometric parameters (Å, º)

**Table:**

C1—N2	1.337 (2)	N23—N24	1.385 (2)
C1—N6	1.347 (2)	N23—C27	1.480 (2)
C1—N23	1.375 (2)	C27—C28	1.505 (3)
N2—C3	1.342 (2)	C27—C26	1.542 (3)
C3—C4	1.396 (3)	C27—H27	0.9800
C3—C7	1.477 (3)	C26—C25	1.498 (3)
C4—C5	1.384 (3)	C26—H26A	0.9700
C4—H4	0.9300	C26—H26B	0.9700
C5—N6	1.346 (2)	C25—N24	1.291 (2)
C5—C15	1.485 (3)	C25—C36	1.465 (3)
C7—C8	1.390 (3)	C28—C29	1.374 (3)
C7—C12	1.393 (3)	C28—C33	1.385 (3)
C8—C9	1.379 (3)	C29—C30	1.386 (3)
C8—H8	0.9300	C29—H29	0.9300
C9—C10	1.377 (3)	C30—C31	1.374 (3)
C9—H9	0.9300	C30—H30	0.9300
C10—O13	1.364 (2)	C31—O34	1.372 (3)
C10—C11	1.380 (3)	C31—C32	1.379 (3)
C11—C12	1.371 (3)	C32—C33	1.374 (3)
C11—H11	0.9300	C32—H32	0.9300
C12—H12	0.9300	C33—H33	0.9300
O13—C14	1.423 (3)	O34—C35	1.411 (3)
C14—H14A	0.9600	C35—H35A	0.9600
C14—H14B	0.9600	C35—H35B	0.9600
C14—H14C	0.9600	C35—H35C	0.9600
C15—C20	1.386 (3)	C36—C41	1.381 (3)
C15—C16	1.397 (3)	C36—C37	1.385 (3)
C16—C17	1.370 (3)	C37—C38	1.382 (3)
C16—H16	0.9300	C37—H37	0.9300
C17—C18	1.378 (3)	C38—C39	1.369 (4)
C17—H17	0.9300	C38—H38	0.9300
C18—O21	1.372 (2)	C39—C40	1.371 (3)
C18—C19	1.376 (3)	C39—O42	1.376 (3)
C19—C20	1.387 (3)	C40—C41	1.379 (3)
C19—H19	0.9300	C40—H40	0.9300
C20—H20	0.9300	C41—H41	0.9300
O21—C22	1.416 (3)	O42—C43	1.414 (3)
C22—H22A	0.9600	C43—H43A	0.9600
C22—H22B	0.9600	C43—H43B	0.9600
C22—H22C	0.9600	C43—H43C	0.9600
			
N2—C1—N6	127.81 (18)	N23—C27—C28	113.01 (16)
N2—C1—N23	117.79 (18)	N23—C27—C26	101.11 (15)
N6—C1—N23	114.39 (17)	C28—C27—C26	114.17 (17)
C1—N2—C3	115.99 (17)	N23—C27—H27	109.4
N2—C3—C4	120.76 (18)	C28—C27—H27	109.4
N2—C3—C7	115.14 (17)	C26—C27—H27	109.4
C4—C3—C7	124.09 (17)	C25—C26—C27	102.96 (16)
C5—C4—C3	118.79 (18)	C25—C26—H26A	111.2
C5—C4—H4	120.6	C27—C26—H26A	111.2
C3—C4—H4	120.6	C25—C26—H26B	111.2
N6—C5—C4	121.15 (18)	C27—C26—H26B	111.2
N6—C5—C15	115.10 (18)	H26A—C26—H26B	109.1
C4—C5—C15	123.74 (18)	N24—C25—C36	120.68 (19)
C5—N6—C1	115.48 (17)	N24—C25—C26	114.29 (18)
C8—C7—C12	116.12 (19)	C36—C25—C26	125.03 (18)
C8—C7—C3	119.82 (18)	C25—N24—N23	107.92 (17)
C12—C7—C3	124.06 (18)	C29—C28—C33	117.68 (19)
C9—C8—C7	122.6 (2)	C29—C28—C27	119.77 (18)
C9—C8—H8	118.7	C33—C28—C27	122.47 (18)
C7—C8—H8	118.7	C28—C29—C30	121.8 (2)
C10—C9—C8	119.8 (2)	C28—C29—H29	119.1
C10—C9—H9	120.1	C30—C29—H29	119.1
C8—C9—H9	120.1	C31—C30—C29	119.4 (2)
O13—C10—C9	124.22 (19)	C31—C30—H30	120.3
O13—C10—C11	116.93 (18)	C29—C30—H30	120.3
C9—C10—C11	118.8 (2)	O34—C31—C30	123.6 (2)
C12—C11—C10	120.9 (2)	O34—C31—C32	116.7 (2)
C12—C11—H11	119.6	C30—C31—C32	119.7 (2)
C10—C11—H11	119.6	C33—C32—C31	120.0 (2)
C11—C12—C7	121.8 (2)	C33—C32—H32	120.0
C11—C12—H12	119.1	C31—C32—H32	120.0
C7—C12—H12	119.1	C32—C33—C28	121.36 (19)
C10—O13—C14	116.95 (18)	C32—C33—H33	119.3
O13—C14—H14A	109.5	C28—C33—H33	119.3
O13—C14—H14B	109.5	C31—O34—C35	117.50 (19)
H14A—C14—H14B	109.5	O34—C35—H35A	109.5
O13—C14—H14C	109.5	O34—C35—H35B	109.5
H14A—C14—H14C	109.5	H35A—C35—H35B	109.5
H14B—C14—H14C	109.5	O34—C35—H35C	109.5
C20—C15—C16	117.00 (19)	H35A—C35—H35C	109.5
C20—C15—C5	120.63 (18)	H35B—C35—H35C	109.5
C16—C15—C5	122.34 (18)	C41—C36—C37	117.3 (2)
C17—C16—C15	121.0 (2)	C41—C36—C25	121.93 (19)
C17—C16—H16	119.5	C37—C36—C25	120.8 (2)
C15—C16—H16	119.5	C38—C37—C36	120.7 (2)
C16—C17—C18	121.3 (2)	C38—C37—H37	119.6
C16—C17—H17	119.4	C36—C37—H37	119.6
C18—C17—H17	119.4	C39—C38—C37	121.0 (2)
O21—C18—C19	125.3 (2)	C39—C38—H38	119.5
O21—C18—C17	115.70 (19)	C37—C38—H38	119.5
C19—C18—C17	119.0 (2)	C38—C39—C40	119.2 (2)
C18—C19—C20	119.7 (2)	C38—C39—O42	116.7 (2)
C18—C19—H19	120.1	C40—C39—O42	124.1 (2)
C20—C19—H19	120.1	C39—C40—C41	119.8 (2)
C15—C20—C19	122.0 (2)	C39—C40—H40	120.1
C15—C20—H20	119.0	C41—C40—H40	120.1
C19—C20—H20	119.0	C40—C41—C36	122.1 (2)
C18—O21—C22	117.49 (18)	C40—C41—H41	119.0
O21—C22—H22A	109.5	C36—C41—H41	119.0
O21—C22—H22B	109.5	C39—O42—C43	118.0 (2)
H22A—C22—H22B	109.5	O42—C43—H43A	109.5
O21—C22—H22C	109.5	O42—C43—H43B	109.5
H22A—C22—H22C	109.5	H43A—C43—H43B	109.5
H22B—C22—H22C	109.5	O42—C43—H43C	109.5
C1—N23—N24	120.71 (17)	H43A—C43—H43C	109.5
C1—N23—C27	123.77 (16)	H43B—C43—H43C	109.5
N24—N23—C27	113.61 (14)		
			
N6—C1—N2—C3	−1.9 (3)	N2—C1—N23—C27	−172.80 (17)
N23—C1—N2—C3	179.00 (17)	N6—C1—N23—C27	8.0 (3)
C1—N2—C3—C4	1.0 (3)	C1—N23—C27—C28	−75.8 (2)
C1—N2—C3—C7	−179.94 (16)	N24—N23—C27—C28	119.86 (18)
N2—C3—C4—C5	−0.1 (3)	C1—N23—C27—C26	161.72 (19)
C7—C3—C4—C5	−179.10 (17)	N24—N23—C27—C26	−2.6 (2)
C3—C4—C5—N6	−0.1 (3)	N23—C27—C26—C25	3.1 (2)
C3—C4—C5—C15	179.57 (18)	C28—C27—C26—C25	−118.58 (19)
C4—C5—N6—C1	−0.5 (3)	C27—C26—C25—N24	−3.1 (3)
C15—C5—N6—C1	179.76 (16)	C27—C26—C25—C36	177.7 (2)
N2—C1—N6—C5	1.6 (3)	C36—C25—N24—N23	−179.24 (18)
N23—C1—N6—C5	−179.23 (17)	C26—C25—N24—N23	1.5 (3)
N2—C3—C7—C8	−0.9 (3)	C1—N23—N24—C25	−164.00 (19)
C4—C3—C7—C8	178.11 (19)	C27—N23—N24—C25	0.8 (2)
N2—C3—C7—C12	179.56 (19)	N23—C27—C28—C29	143.75 (19)
C4—C3—C7—C12	−1.4 (3)	C26—C27—C28—C29	−101.4 (2)
C12—C7—C8—C9	0.7 (3)	N23—C27—C28—C33	−39.5 (3)
C3—C7—C8—C9	−178.9 (2)	C26—C27—C28—C33	75.3 (2)
C7—C8—C9—C10	−0.9 (4)	C33—C28—C29—C30	−0.4 (3)
C8—C9—C10—O13	179.4 (2)	C27—C28—C29—C30	176.5 (2)
C8—C9—C10—C11	1.0 (3)	C28—C29—C30—C31	0.0 (4)
O13—C10—C11—C12	−179.4 (2)	C29—C30—C31—O34	179.3 (2)
C9—C10—C11—C12	−0.9 (3)	C29—C30—C31—C32	0.4 (4)
C10—C11—C12—C7	0.7 (3)	O34—C31—C32—C33	−179.5 (2)
C8—C7—C12—C11	−0.5 (3)	C30—C31—C32—C33	−0.5 (3)
C3—C7—C12—C11	179.02 (19)	C31—C32—C33—C28	0.2 (3)
C9—C10—O13—C14	−2.3 (3)	C29—C28—C33—C32	0.3 (3)
C11—C10—O13—C14	176.2 (2)	C27—C28—C33—C32	−176.56 (19)
N6—C5—C15—C20	−9.0 (3)	C30—C31—O34—C35	0.5 (4)
C4—C5—C15—C20	171.3 (2)	C32—C31—O34—C35	179.4 (3)
N6—C5—C15—C16	169.18 (19)	N24—C25—C36—C41	−10.5 (3)
C4—C5—C15—C16	−10.5 (3)	C26—C25—C36—C41	168.6 (2)
C20—C15—C16—C17	1.0 (3)	N24—C25—C36—C37	168.4 (2)
C5—C15—C16—C17	−177.3 (2)	C26—C25—C36—C37	−12.5 (3)
C15—C16—C17—C18	0.1 (4)	C41—C36—C37—C38	0.4 (4)
C16—C17—C18—O21	178.4 (2)	C25—C36—C37—C38	−178.5 (3)
C16—C17—C18—C19	−1.2 (4)	C36—C37—C38—C39	−0.5 (5)
O21—C18—C19—C20	−178.5 (2)	C37—C38—C39—C40	0.2 (5)
C17—C18—C19—C20	1.1 (4)	C37—C38—C39—O42	−179.0 (3)
C16—C15—C20—C19	−1.0 (3)	C38—C39—C40—C41	0.2 (4)
C5—C15—C20—C19	177.3 (2)	O42—C39—C40—C41	179.4 (3)
C18—C19—C20—C15	0.0 (4)	C39—C40—C41—C36	−0.3 (4)
C19—C18—O21—C22	3.8 (3)	C37—C36—C41—C40	0.0 (4)
C17—C18—O21—C22	−175.8 (2)	C25—C36—C41—C40	178.9 (2)
N2—C1—N23—N24	−9.5 (3)	C38—C39—O42—C43	177.1 (3)
N6—C1—N23—N24	171.21 (17)	C40—C39—O42—C43	−2.1 (4)

## References

[bb1] Baktır, Z., Akkurt, M., Samshuddin, S., Narayana, B. & Yathirajan, H. S. (2011). *Acta Cryst.* E**67**, o1262–o1263. 10.1107/S1600536811015455 PMC3089231 21754550

[bb2] Betz, R., Gerber, T., Hosten, E., Samshuddin, S., Narayana, B. & Sarojini, B. K. (2012). *Acta Cryst.* E**68**, o476–o477. 10.1107/S1600536812001912 PMC3275226 22347082

[bb3] Calabresi, P., Parks, R. E., Goodman, L. S. & Gilman, A. (1975). *The Pharmacological Basis of Therapeutics*, 5th ed., p. 1254. New York: Macmillan.

[bb4] El-Hashash, M. A., Mahmoud, M. R. & Madboli, S. A. (1993). *Indian J. Chem. Sect. B*, **32**, 449–451.

[bb5] Farrugia, L. J. (1997). *J. Appl. Cryst.* **30**, 565.

[bb6] Fun, H.-K., Chia, T. S., Samshuddin, S., Narayana, B. & Sarojini, B. K. (2012). *Acta Cryst.* E**68**, o807–o808. 10.1107/S1600536812006976 PMC3297871 22412674

[bb7] Fun, H.-K., Hemamalini, M., Samshuddin, S., Narayana, B. & Yathirajan, H. S. (2010). *Acta Cryst.* E**66**, o582–o583. 10.1107/S1600536810004435 PMC2983722 21580348

[bb8] Jasinski, J. P., Guild, C. J., Samshuddin, S., Narayana, B. & Yathirajan, H. S. (2010). *Acta Cryst.* E**66**, o1948–o1949. 10.1107/S1600536810026036 PMC3007318 21588274

[bb9] Oxford Diffraction (2010). *CrysAlis PRO* Oxford Diffraction Ltd, Yarnton, Oxfordshire, England.

[bb10] Samshuddin, S., Narayana, B., Shetty, D. N. & Raghavendra, R. (2011). *Pharm. Chem.* **3**, 232–240.

[bb11] Sheldrick, G. M. (2008). *Acta Cryst.* A**64**, 112–122. 10.1107/S0108767307043930 18156677

[bb12] Spek, A. L. (2009). *Acta Cryst.* D**65**, 148–155. 10.1107/S090744490804362X PMC2631630 19171970

